# Supplementary material for: Collaborative intelligence in AI: Evaluating the performance of a council of AIs on the USMLE
Source: PLOS Digit Health. 2025 Oct 9;4(10):e0000787. doi: 10.1371/journal.pdig.0000787 (PMC12510544; doi:10.1371/journal.pdig.0000787)
Supplement: S2 File — (ZIP) [file pdig.0000787.s002.zip › S2 Data Raw data files containing unprocessed question inputs and LLM outputs including transcripts of deliberations/STEP3_Question-46-1_Type-MC-NJ.docx]

**Facilitator** **[Round 1]:** A 33-year-old male physician reports for a shift in the emergency department. A nurse alerts you that he noticed a faint odor of alcohol near the physician. When approached, the physician appears tired and more disheveled than usual. There is an odor of alcohol on his breath. He is a skilled and talented physician with no known history of substance or alcohol use disorder. He is married with three children, and his wife is pregnant with twins. The physician was recently hired and has had no actions on his license by any state medical board. Which of the following is the most appropriate next step? (A) Ask the physician if he is sober, and if he says yes, allow him to complete his shift (B) Explain to the physician that you suspect he is intoxicated and ask him to submit to a blood sample to check his blood alcohol concentration (C) Relieve the physician of duty and alert the hospital's patient safety officer (D) Tell the physician that you can cover the remainder of the shift alone, so that he can sleep in his office (E) Tell the physician you detect alcohol on his breath, and he needs to go home and should not return until he is sober

**AI_C:** RESTATING THE QUESTION: The provided scenario describes a situation in which a 33-year-old male physician, who has recently started working and historically exhibits dedicated professional behavior, appears at his emergency department shift with an unusual appearance. Observations include him appearing tired and disheveled, and an odor of alcohol can be detected on his breath. The question asks, what would be the most appropriate action to take in response to these observations? PLANNING: Main Assumption: The physician is under the influence of alcohol, which affects his ability to provide safe and effective care. Alternate Assumption 1: The physician's physical changes are due to other factors such as exhaustion or stress, leading to mistaken identification of an alcohol-odor. Alternate Assumption 2: The physician is under the influence of alcohol, but he is fully competent to perform his duties. Critical Assumption: The presence of alcohol odor is indicative of alcohol consumption, which could impair the physician's professional duties. CLINICAL REASONING: The core ethical and professional value in practicing medicine is ensuring the safety of patients. It's widely understood that alcohol impairs cognition, psychomotor function, and decision-making abilities, potentially endangering patients' health. Even if the physician denies alcohol consumption or asserts his capability to work despite consumption, the mere perception of a potential risk is significant. Given the physician's generally professional demeanor, it's conceivable that stress or fatigue could produce perceived disheveled appearance and possible smell of alcohol. However, this doesn't lessen the potential risk to patients, especially in an emergency department setting where quick decision-making and precision are vital. CONCLUDING: Given the potential risk to patient safety, the priority is to ensure the physician is sober before treating patients. Therefore, the immediate relief from duty is essential until clarity can be obtained on the situation. While a blood sample could be useful, it might invade personal rights and require consent. It's also crucial to alert the patient safety officer to maintain transparency and address the situation appropriately. RESPONSE: The most appropriate next step in this scenario is (C) Relieve the physician of duty and alert the hospital's patient safety officer. This reduces immediate risk to patient care while allowing time to properly address the potential issue. It also maintains professional standards and safeguards hospital reputation.

**AI_A:** 1) RESTATING THE QUESTION: The scenario here concerns a 33-year-old male physician who has arrived for a shift in the ER, seeming disheveled and exhausted, and with a noticeable alcohol odor on his breath. Despite his recognised competence and a clean professional history, there is cause for concern due to these alarming indicators. Given this situation, we are required to determine the most suitable course of action from various alternatives provided. 2) PLANNING: The primary assumption here is that his unusual appearance and the alcohol odor are signs of negligence, which can endanger patients' lives. This assumption leads us toward options that involve confrontation or reporting. However, we can alternatively assume that some other factors, perhaps personally stressful events, have led him to this state, requiring an approach embodying support and guidance. The latter alternate assumption bears weight given he has personal pressures and no substance misuse history, pushing us toward an action plan that is instant yet considerate, without jeopardizing patient safety. 3) CLINICAL REASONING: As health professionals, our chief obligation is ensuring our patients' safety. Even if one is a skilled and talented physician, impairment due to substance use can potentially harm patients. Conversely, he has no known history of substance misuse. His wife's twin pregnancy, and their three children could imply stressful personal circumstances. On balance of these elements, it is plausible that he could have resorted to alcohol as a coping mechanism. This is unusual but not surprising, given that health professionals are known to experience considerable stress. However, this doesn't exempt the physician from potential consequences – intoxicated doctors could commit errors leading to patient harm. 4) CONCLUDING: Maintaining patient safety and upholding the hospital's ethical standard warrant immediate action. Yet, an affable and supportive gesture toward the physician, as a colleague undergoing potential personal difficulty, is crucial. Detecting sobriety by asking him serves no productive purpose and relies on self-report. A blood-alcohol test does evaluate sobriety accurately but can be overly invasive and might strain professional relationships. Proposing he sleep in his office or return when sober may unintentionally sanction his current unacceptable state. 5) RESPONSE: Hence, the most suitable step would be to (C) Relieve the physician of his duty and alert the hospital's patient safety officer, ensuring patient well-being, and propelling appropriate inquiry into the matter without passing premature judgment or enforcing immediate punitive measures. It enables the hospital administration to carry out more discreet and comprehensive evaluations to understand if this is a one-off instance or part of an emerging, detrimental pattern that can affect his professional run.

**AI_E:** RESTATING THE QUESTION: We are faced with a situation where there is suspicion of a physician, new to the hospital, possibly reporting for duty under the influence of alcohol. The signs suggesting this suspicion include a disheveled appearance, a faint smell of alcohol around him and alcohol-connoted breath upon close approach. Even though he is highly skilled and has no history of substance misuse, his current state may pose concerns about patient safety. Given this situation, we must decide the most appropriate next course of action which include options like querying him about his sobriety, asking for a blood alcohol concentration test, relieving him from duty and alerting the safety officer, or suggesting he sleeps it off in the office or going home until sober. PLANNING: This situation highlights a potential risk to patient safety as a possibly impaired physician could make critical errors. Additionally, the physician's actions could potentially violate professional standards and ethical expectations. Though there's no prior history of substance misuse, it's essential not to assume his competent history rules out the possibility of current intoxication. Assumptions also can not be made based on personal situations. The physician might be under stress due to personal (family expectations) or professional reasons (new job), and these stressors may or may not be related to this situation. CLINICAL REASONING: In any case where a physician may potentially be impaired, the primary focus must always be on patient safety. Assuming sobriety based on his word or professionalism without rest, isn't a reliable plan as people under influence may be unaware or deny their altered state. Option of allowing him to sleep off in the office may not fully solve the problem, as it does not address the potential risk he may pose when he resumes work. On the other hand, suggesting that he go home until sober also does not address the possible implications of his actions or the evaluation of the reason for his current state. Requesting a blood alcohol concentration test is invasive, and there could be potential legal implications as well. CONCLUDING: The most appropriate step should ensure patient safety without acknowledging or assuming the cause of the physician's change in state. It should present a safe and respectful way to remove the physician from a potentially hazardous situation, have a formal way to address it and invite an investigation into the event without preconceived judgement. RESPONSE: Based on these elements, the preferred course of action in this scenario would be option (C) Relieve the physician of duty and alert the hospital's patient safety officer. This action ensures immediate patient safety and allows an impartial party to conduct a thorough and appropriate investigation without directly accusing the physician of being under the influence at the outset.

**AI_D:** RESTATING THE QUESTION: The scenario presents a 33-year-old male physician who seems to be under the influence of alcohol on-duty, as indicated by the odor detected by a nurse and his overall disheveled appearance. His personal background includes a stable family life and a clean professional record, and he shows no prior history of any substance abuse issues. The question is asking what is the most appropriate action to take under these circumstances. These options range from permitting him to carry on with his shift on his confirmation of sobriety, to asking him to submit to a blood sample for alcohol testing, to removing him from duty and informing the patient safety officer, to allowing him to sleep it out in his office, or to asking him to go home until he is sober. PLANNING: The premises in the presented case are the signs pointing to this physician's possible state of intoxication. Alcohol intoxication can affect cognitive processes and motor skills, both crucial for a physician, especially working in an emergency department. Even though this physician has no known history of substance use disorder, it is crucial to prioritize patient safety. Main assumptions could be that the observed signs are due to acute alcohol intake or to other issues such as fatigue or stress. Alternately, one might assume the physician would not be impaired enough to affect patient care. CLINICAL REASONING: We are dealing with a possibly intoxicated physician in an ED setting. All options need to be evaluated while keeping patient safety paramount. Option A leaves room for a false negative if the physician isn't sober but says he is. Option B is confrontational, potentially damaging the professional relationship. It focuses on determining intoxication rather than dealing with its potential immediate hazards. Option D overtly neglects the issue's seriousness and might indirectly enable a dangerous precedent. Option E, while proactive, could also be seen as punitive and confrontational. CONCLUDING: The best option should prioritize patient safety without risking or damaging professional relationships. An episode of intoxication, especially in a person with no previous history, might be indicative of increased stress, perhaps related to personal life changes or professional adjustment. Crucially, it should approach the issue proactively but non-punitively and guide the physician towards addressing any possible underlying cause of such behavior. RESPONSE: Considering all these factors I deem 'Option C' to be the most appropriate next course of action - "Relieve the physician of duty and alert the hospital's patient safety officer". This would ensure avoiding any potential hazard to patients, provide some relief to the physician, and also involve relevant institutional authority to follow due process and ensure a thorough, considerate evaluation of the physician's situation.

**AI_B:** RESTATING THE QUESTION: So, the notable issue here is regarding a 33-year-old male physician who has just reported for his shift but allegedly has an alcohol odor. He appears more disheveled and fatigued than is typical for him. Even though he has no recorded history of alcohol or substance abuse, he currently appears to be in a state that could be concerning. He has no previous actions on his medical license. The main query is to figure out the most appropriate course of action under these circumstances. The alternatives given range from allowing him to proceed with his shift, having him provide a blood sample for alcohol concentration analysis, relieving him of his duties and alerting the patient safety officer, offering to cover for him so he can rest, or informing him about the detected alcohol and ask him to leave and resume work only when he's completely sober. PLANNING: The first assumption is that the physician is intoxicated on the job, a serious violation that can put patients' lives at risk. One alternative assumption here could be that he is possibly facing a personal issue, leading to a change in his demeanor and possibly the consumption of alcohol before his duty. Another assumption could be his potential struggle with alcoholism, despite a lack of any recorded history. We need to evaluate which assumption(s) hold true and what should be the priority clinically that would not compromise patient safety. CLINICAL REASONING: Given the possibility of potentially intoxication, various factors need consideration. Approaching the issue professionally and ethically is paramount. The significant smell of alcohol, his disheveled appearance, and the likely inebriation require immediate attention. Inquiring about his sobriety (Option A) might not warrant reliable answers, especially when the physician may fear job security. Asking for an immediate blood alcohol concentration check might be confrontational and evoke defensiveness (Option B). Covering his shift and allowing him to rest in the office (Option D) doesn't appropriately address the alleged alcohol issue. Similarly, sending him back home (Option E) without probing the issue further or taking constructive action seems inappropriate as it bypasses the responsibility of addressing the issue head-on. CONCLUDING: In this scenario, the priority should be to guarantee that the quality of care and patient safety isn't compromised. It's crucial to wisely and compassionately handle the situation without making any assumptions about the physician's character or personal life. From a clinical standpoint, the symptoms point out to possible intoxication which can harm patients' safety. RESPONSE: Hence, the most appropriate next step would be to (C) Relieve the physician of duty and alert the hospital's patient safety officer. This ensures immediate mitigating action to ensure patient safety and initiates a process of understanding the physician's issues, without any undue personal or professional embarrassment. Further, action can be taken to understand if it is a one-time scenario due to personal issues or a symptom of a more severe problem like alcohol use disorder.

**Facilitator** The consensus among the AI responses is that the most appropriate course of action in the given scenario, where a 33-year-old male physician appears disheveled and has a noticeable alcohol odor on his breath, is to relieve the physician of his duty and alert the hospital's patient safety officer. This decision is based on the primary assumption that the physician's unusual appearance and the alcohol odor are signs of potential impairment, which could endanger patients' lives. Despite the physician's recognized competence and clean professional history, patient safety is the paramount concern. The responses also consider an alternate assumption that personal stressors, such as his wife's twin pregnancy and their three children, may have led him to this state. However, even if this is the case, it does not lessen the potential risk to patients. The responses reject other options such as asking the physician about his sobriety, requesting a blood-alcohol test, proposing he sleep in his office, or asking him to return when sober, as these either rely on self-report, could strain professional relationships, or may unintentionally sanction his current unacceptable state. By choosing to relieve the physician of his duty and alert the hospital's patient safety officer, immediate action is taken to ensure patient safety, while also initiating a process to understand the physician's issues without any undue personal or professional embarrassment. This approach allows for a more discreet and comprehensive evaluation to determine if this is a one-off instance or part of an emerging, detrimental pattern that could affect his professional performance.
